# Chemosensitizing activity of peptide from *Lentinus squarrosulus* (Mont.) on cisplatin-induced apoptosis in human lung cancer cells

**DOI:** 10.1038/s41598-021-83606-1

**Published:** 2021-02-18

**Authors:** Hnin Ei Ei Khine, Gea Abigail Uy Ecoy, Sittiruk Roytrakul, Narumon Phaonakrop, Natapol Pornputtapong, Eakachai Prompetchara, Pithi Chanvorachote, Chatchai Chaotham

**Affiliations:** 1 Department of Biochemistry and Microbiology, Faculty of Pharmaceutical Sciences, Chulalongkorn University, Bangkok, 10330 Thailand; 2 Department of Pharmacy, School of Health Care Professions, University of San Carlos, 6000 Cebu, Philippines; 3 Functional Ingredients and Food Innovation Research Group, National Center for Genetic Engineering and Biotechnology (BIOTEC), Pathum Thani, 12120 Thailand; 4 Center of Excellence in Systems Biology, Faculty of Medicine, Chulalongkorn University, Bangkok, 10330 Thailand; 5 Department of Laboratory Medicine, Faculty of Medicine, Chulalongkorn University, Bangkok, 10330 Thailand; 6 Center of Excellence in Vaccine Research and Development (Chula Vaccine Research Center-Chula VRC), Faculty of Medicine, Chulalongkorn University, Bangkok, 10330 Thailand; 7 Department of Pharmacology and Physiology, Faculty of Pharmaceutical Sciences, Chulalongkorn University, Bangkok, 10330 Thailand; 8 Cell-Based Drug and Health Products Development Research Unit, Faculty of Pharmaceutical Sciences, Chulalongkorn University, Bangkok, 10330 Thailand

**Keywords:** Molecular medicine, Drug development

## Abstract

The limitations of cisplatin, a standard chemotherapy for lung cancer, have been documented with serious adverse effects and drug resistance. To address the need for novel therapy, this study firstly reveals the potential of peptide from *Lentinus squarrosulus* (Mont.) as a chemotherapeutic adjuvant for cisplatin treatment. The purified peptide from *L. squarrosulus* aqueous extracts was obtained after eluting with 0.4 M NaCl through FPLC equipped with anion exchange column. Preincubation for 24 h with 5 µg/mL of the peptide at prior to treatment with 5 µM cisplatin significantly diminished %cell viability in various human lung cancer cells but not in human dermal papilla and proximal renal cells. Flow cytometry indicated the augmentation of cisplatin-induced apoptosis in lung cancer cells pretreated with peptide from *L. squarrosulus*. Preculture with the peptide dramatically inhibited colony formation in lung cancer cells derived after cisplatin treatment. Strong suppression on integrin-mediated survival was evidenced with the diminution of integrins (β1, β3, β5, α5, αV) and down-stream signals (p-FAK/FAK, p-Src/Src, p-Akt/Akt) consequence with alteration of p53, Bax, Blc-2 and Mcl-1 in cisplatin-treated lung cancer cells preincubated with peptide from *L. squarrosulus*. These results support the development of *L. squarrosulus* peptide as a novel combined chemotherapy with cisplatin for lung cancer treatment.

## Introduction

Despite several decades of intensive research, lung cancer remains to be a global health burden and the leading cause of cancer-related death^1^. Among various available remedies, standard chemotherapy is still one of the major treatment options due to its known efficacy and relatively good patient compliance^2^. Cisplatin, a platinum-based compound, is a first-line chemotherapeutic drug for the treatment of lung cancer^3^. In combination with other drugs, it is commonly used as adjuvant therapy for the advanced stages of lung cancer and after tumor resection^4^. However, the aggressive features and distinct mechanisms observed in lung cancer cells lead to chemotherapeutic resistance and treatment failure^5,6^. Moreover, adverse effects such as renal toxicity and alopecia further compromise patients’ quality of life and limit the use of cisplatin^7^. To improve compliance and clinical outcomes, a significant amount of research has been conducted to find novel therapies to restore cisplatin sensitivity in cancer cells without increasing toxicity to normal cells^8,9^.

While the aim is to maximize cisplatin efficacy, evidence shows that cisplatin resistance is associated with reduced apoptosis induction in lung cancer cells^10^. Current research trends reveal that targeting Bcl-2 family proteins is a promising strategy to overcome cisplatin resistance^11,12^. The upregulation or overexpression of the anti-apoptosis protein, Bcl-2 (B-cell lymphoma 2), is related to the reduced susceptibility to cisplatin-induced apoptosis^10,13^. Moreover, evidence suggests that the upregulation of another anti-apoptosis protein, Mcl-1 (Myeloid cell leukemia 1) is correlated to the persistence of tumor pathology in lung cancer patients^14^. On the other hand, the downregulation of Mcl-1 protein has been found to effectively restore drug sensitivity in cisplatin-resistant lung cancer cells^15^. Meanwhile, the decreased protein level of pro-apoptosis Bax (Bcl-2 associated X) protein causes hyperplasia of lung tumor and resistance to various apoptosis stimuli^16^. Evidently, the modulation of Bcl-2 family proteins to reverse cisplatin resistance is worth investigating further. Another strategy is the regulation of tumor suppressor p53 which has been a reported mechanism of several natural chemosensitizers^9^.

Dysregulation of survival signaling pathways has also been previously linked to cisplatin resistance. Elevated level of FAK (Focal adhesion kinase) also protects lung cancer cells from cisplatin-induced apoptosis^17^. The complexation between transmembrane molecules, integrins, and extracellular matrix (ECM) initiates the survival cascade via stimulation of FAK/Src (Proto-oncogene tyrosine-protein kinase) consequently activating the downstream PI3K (phosphoinositide 3-kinase)/Akt (Protein kinase B) signaling pathway^18^. The overexpression of integrins especially β1, β3, β5, α5 and αV, have particularly been implicated in lung cancer progression and cisplatin treatment failure^19–21^. In addition, the upregulation of PI3K/Akt-related survival molecules by integrin-ECM interaction results in the activation of anti-apoptosis proteins, inhibition of p53 and disruption of apoptosis cell death^22,23^. Therefore, the regulation of integrins and corresponding survival molecules has emerged as another strategy to augment cisplatin potency^24–26^.

Due to the obvious need of innovation in cancer treatment, multi-targeted natural chemosensitizers used in combination with standard anticancer drugs have recently been highlighted for their capacity to enhance efficacy and reduce toxicities of chemotherapy^9,27^. Among various natural resources, peptide extracts from edible mushroom have gained an attention due to anti-tumor potential and promising safety profile^28^. Recently, peptides extracted from *Lentinus squarrosulus* (Mont.) have been revealed to induce apoptosis in human lung cancer cells^29^. However, the reported extracts composed of various peptides ranging from 11 to 75 kDa which may contain some toxicity. In order to improve efficacy of these peptide extracts, the purification of *L. squarrosulus* peptide extracts have been performed through fast protein liquid chromatography (FPLC) in this study. Furthermore, this study is the first to investigate the novel chemosensitizing activity of the peptide extracted from *L. squarrosulus* to enhance cisplatin efficacy without causing toxicity to non-cancer cells. The investigation seeks to provide information which could encourage the further development of chemotherapeutic adjuvants with good safety profile for lung cancer treatment.

## Results

### Purified peptide isolated from *L. squarrosulus* extracts

**Figure 1:**
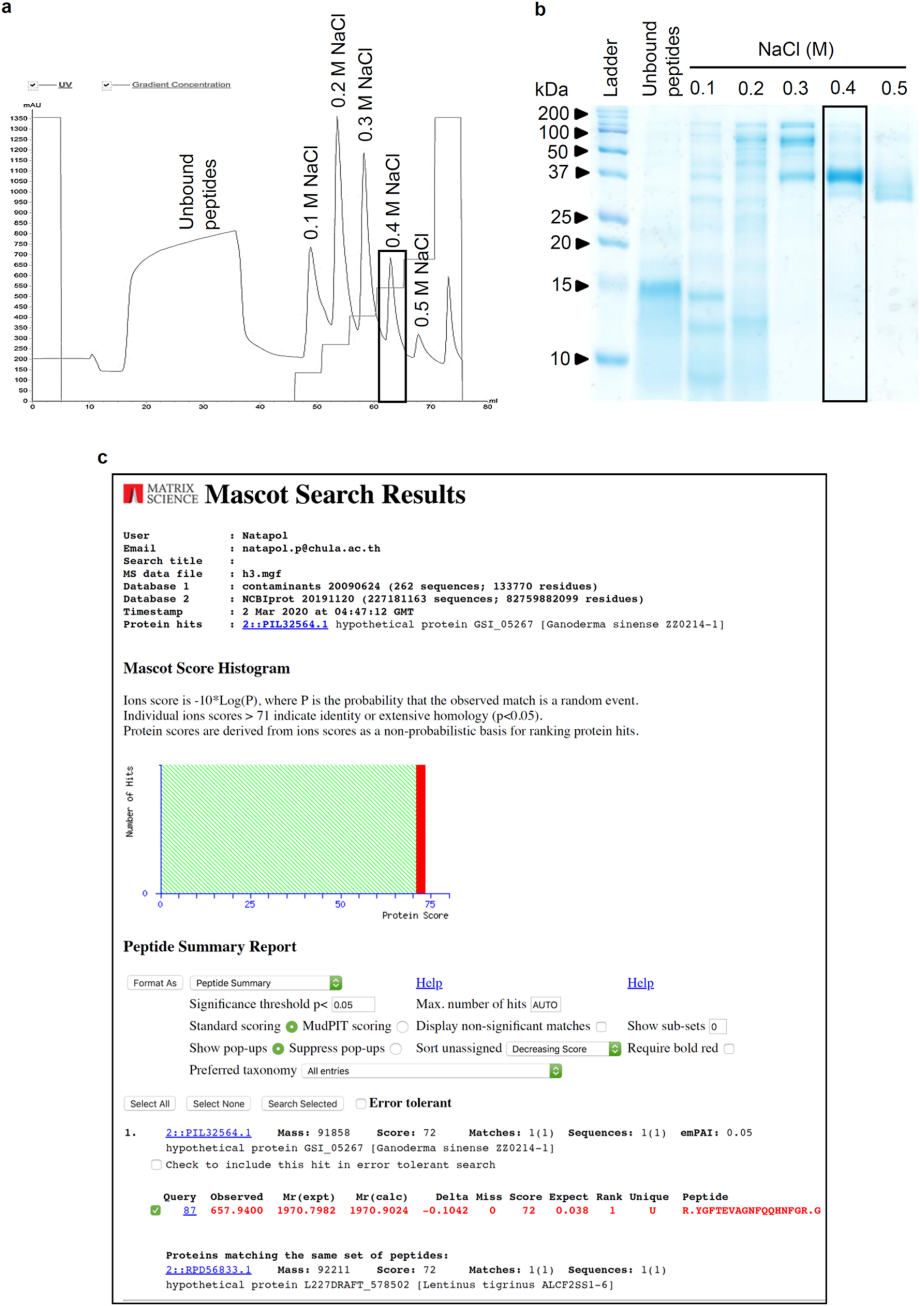
Purified peptide from aqueous extract of *Lentinus squarrosulus* (Mont.). (**a**) Chromatogram of eluted peptides obtained from FPLC at different concentrations of NaCl. (**b**) Peptide composition of different fractions from *L. squarrosulus* extracts analyzed by SDS-PAGE. The rectangular box indicates the purest fraction, 0.4 M NaCl peptide. (**c**) The Mascot search web service indicated 72 similarity score of proteomic analysis of 0.4 M NaCl peptide with extracellular metalloproteinase from *Ganoderma sinense* ZZ0214-1 and *Lentinus tigrinus* ALCF2SS1-6.

The peptide pellets were precipitated out from the homogenized aqueous solution of *L. squarrosulus* fruiting bodies by adding 40–80% (NH_4_)_2_SO_4_. These crude peptide extracts were then resolubilized in PBS (pH 7.4) and further purified through FPLC coupled with anion exchange column. Figure 1a shows a representative FPLC chromatogram of different peptide fractions which were eluted through a stepwise increase in concentration of NaCl solution (0.1, 0.2, 0.3, 0.4 and 0.5 M). The eluted peptide profile was composed of six peaks including unbound peptides. The composition of each peptide fraction was evaluated through SDS-PAGE analysis. Among the six peptide fractions, the most purified peptide appears in the fraction eluted with 0.4 M NaCl, which presented an intensively stained single band of protein at ~ 37 kDa (Fig. 1b). It is worth noting that protein bands appearing with less intensity and various molecular weight were presented in the other peptide fractions. Therefore, the peptide of 0.4 M NaCl fraction was selected for further investigations on chemosensitizing effect to cisplatin.

### Proteomics analysis of *L. squarrosulus* purified fraction

Despite hundreds of peptide fragments separated through LC–MS/MS, the search results reported only one matched query. Fragments with observed mass at 657.9400 were uniquely matched (*p* < 0.05) with the peptide sequence (R.YGFTEVAGNFQQHNFGR.G) from hypothetical protein GSI_05267 of *Ganoderma sinense* ZZ0214-1 and the same set of peptides was also matched to hypothetical protein L227DRAFT_578502 of *Lentinus tigrinus* ALCF2SS1-6 as shown in Fig. 1c. The peptide sequence of matched fragments was then 100% identity matched to “A0A2G8SFL4_9APHY—Extracellular metalloproteinase *Ganoderma sinense* ZZ0214-1” and “A0A5C2S0B7_9APHY—Extracellular metalloproteinase *Lentinus tigrinus* ALCF2SS1-6” from UniProt database.

### Cytotoxicity of *L. squarrosulus* peptide

**Figure 2:**
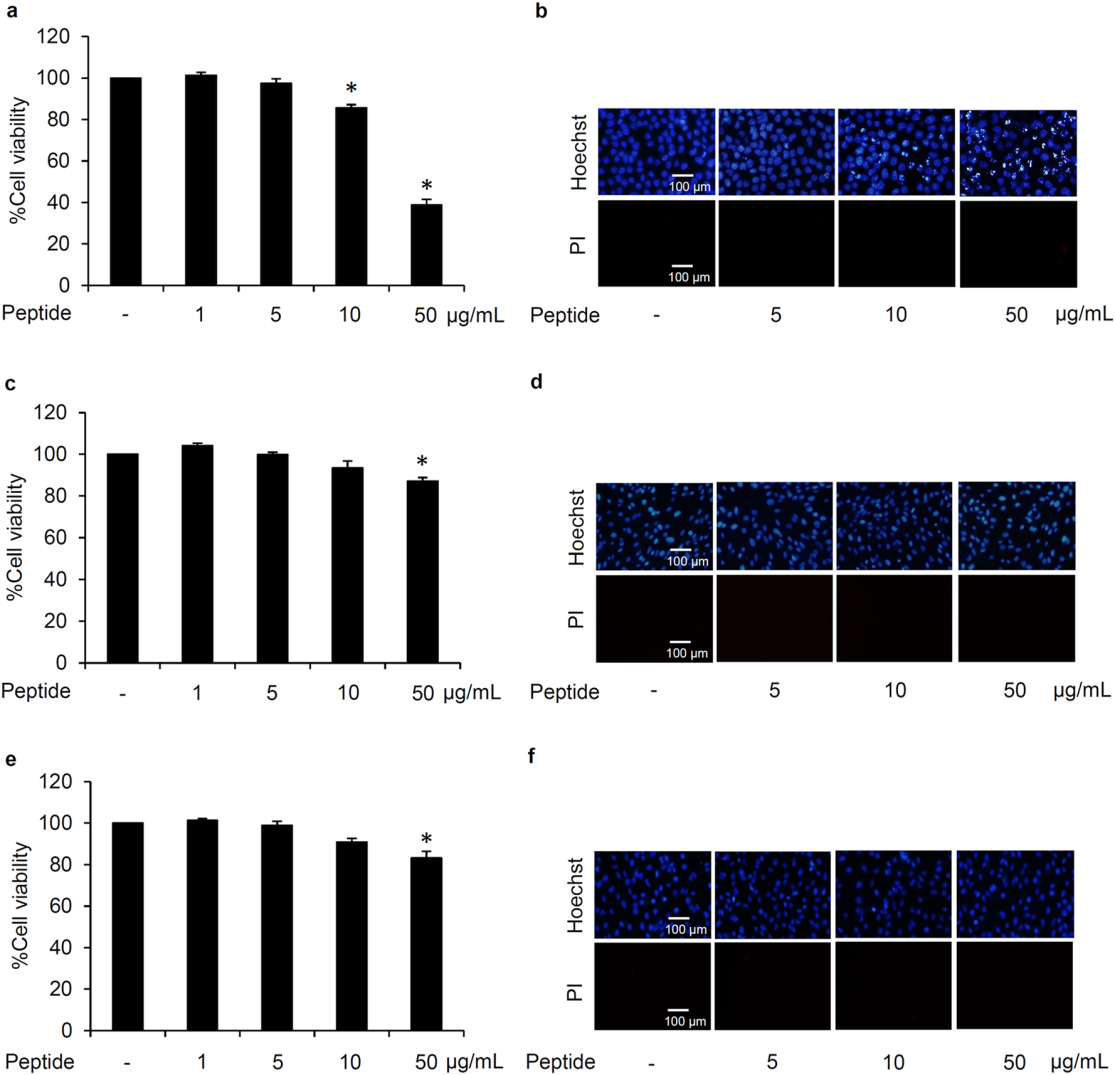
Cytotoxicity profile of *L. squarrosulus* peptide from 0.4 M NaCl fraction in human lung cancer and normal cells. The non-toxic concentration range of the peptide was determined in (**a**) human lung cancer H460, (**c**) dermal papilla DPCs, and (**e**) proximal renal HK-2 cells cultured with 0.4 M NaCl peptide (0–50 µg/mL) for 24 h via MTT viability assay. Costaining with Hoechst33342 and propidium iodide (PI) indicated no detectable apoptosis and necrosis (**b**) H460, (d) DPCs, and (f) HK-2 cells after treatment with 5 µg/mL of 0.4 M NaCl peptide for 24 h. Notably, no cell death was observed in DPCs and HK-2 cells at higher concentrations of the peptide while apoptosis cells were visually in Hoechst33342-stained H460 cells cultured with 10–50 µg/mL of 0.4 M NaCl peptide. Values are means of three independent experiments ± SD. *p < 0.05 versus non-treated control cells.

To clarify the optimum concentrations for further use in the study, the cytotoxic effect of 0.4 M NaCl *L. squarrosulus* peptide fraction was investigated in both human lung cancer and normal cells. After incubation with various concentrations (0–50 µg/mL) of 0.4 M NaCl peptide for 24 h, the reduction of %cell viability was significantly noted in human lung cancer H460 cells treated with 10 and 50 µg/mL of the peptide (Fig. 2a). Corresponding with the viability assay results, apoptosis as characterized by the bright blue fluorescence of Hoechst33342 nuclear stain was evidently increased in the cells cultured with 10–50 µg/mL of 0.4 M NaCl peptide (Fig. 2b). Notably, there were no red fluorescence cells signifying necrosis in all peptide-treated lung cancer cells.

Because hair loss and renal failure are major side effects of cisplatin^30–32^, the cytotoxicity of 0.4 M NaCl peptide was also investigated in human dermal papilla DPCs cells and human proximal renal HK-2 cells. Figure 2c,e depict the viability in DPCs and HK-2 cells as determined through MTT assay. The %cell viability remained approximately 100% in both cell types after treatment with 0–10 µg/mL of *L. squarrosulus* peptide for 24 h. Although significant reduction of cell viability was detected, apoptosis and necrosis were barely observed in DPCs and HK-2 cells cultured with peptide of 0.4 M NaCl fraction at 50 µg/ml for 24 h (Fig. 2d,f). Remarkably, the peptide extract possesses selective cytotoxicity towards lung cancer cells which is evident in the lower %cell viability and increased apoptosis observed in human lung cancer H460 cells after treatment with 0.4 M NaCl peptide at 10–50 µg/ml compared with both DPCs and HK-2 cells.

To clearly clarify chemosensitizing effect, pretreatment of cancer cells with chemotherapeutic adjuvant at non-toxic concentration is suggested^33^. Therefore, 5 µg/mL of 0.4 M NaCl peptide which did not exhibit cytotoxicity in cancer and non-cancer cells was selected as the optimum non-toxic concentration for further experiments related to sensitization to cisplatin-induced cell death in human lung cancer cells.

### *L. squarrosulus* peptide sensitizes cisplatin-induced apoptosis in human lung cancer cells

**Figure 3:**
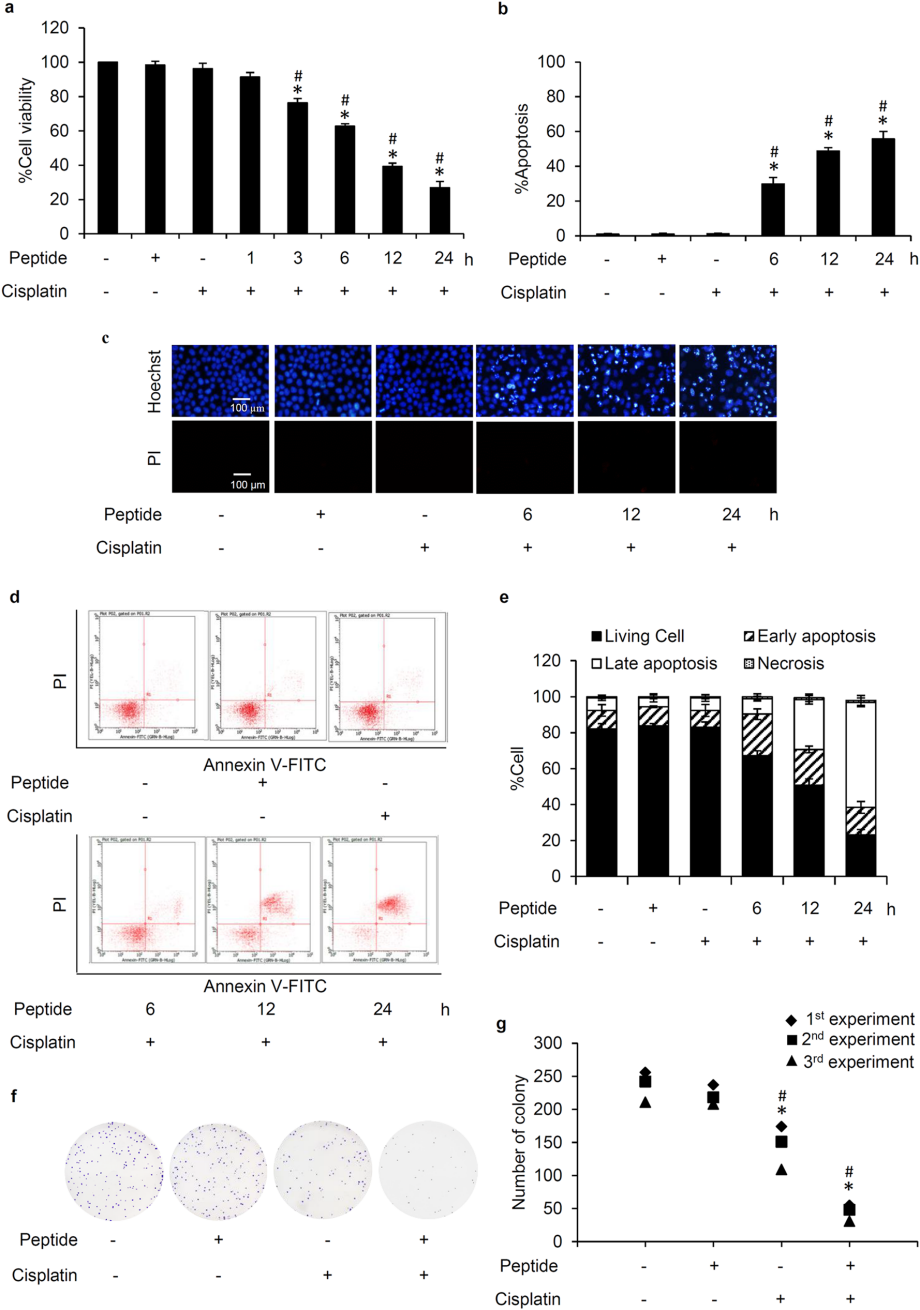
Sensitizing effect of *L. squarrosulus* peptide on cisplatin-induced cytotoxicity in human lung cancer H460 cells. (**a**) The reduction of viability in cisplatin-treated H460 cells was observed in time-dependent manner in response to 0.4 M NaCl peptide at 5 µg/mL. (**b**) Higher %apoptosis detected by (**c**) bright blue fluorescence of Hoechst33342 staining was demonstrated in H460 cells preincubated with the peptide at 5 µg/mL for 6–24 h compared with the cells treated with cisplatin alone. Notably, there was no necrosis characterized by propidium iodide (PI) red fluorescence observed in all treated cells. (**d**) Flow cytometry histograms of annexin V-FITC/PI showed the augmentation of apoptosis in H460 cells preincubated with 0.4 M NaCl peptide (5 µg/mL) then treated with 5 µM of cisplatin for 24 h. (**e**) The higher %cells in both early and late apoptosis was noted in the cells pretreated with *L. squarrosulus* peptide for 6–24 h compared with the cells treated only with cisplatin. Pretreatment with 0.4 M NaCl peptide suppresses colony formation of human lung cancer cells derived after cisplatin treatment. (**f**) Capability to form new colony of lung cancer H460 cells was evaluated via clonogenic assay. (**g**) Lower number of forming colony was demonstrated in cisplatin treated-H460 cells that were precultured with 5 µg/mL of 0.4 M NaCl fraction from *L. squarrosulus* peptides compared with both non-treated control and only cisplatin treated groups. It was worth noting that treatment alone with 0.4 M NaCl peptide did not alter colony formation in lung cancer H460 cells which confirms the non-toxic effect of *L. squarrosulus* peptide at 5 µg/mL. Values shown are means of three independent experiments ± SD. *p < 0.05 versus non-treated control cells, ^#^*p* < 0.05 versus the cells only treated with cisplatin.

The chemosensitizing effect of *L. squarrosulus* peptide was determined in time-dependent investigation in human lung cancer cells. Lung cancer H460 cells were pretreated with 0.4 M NaCl peptide prior to exposure of 5 µM cisplatin. The cell viability of H460 cells further decreased in a time-dependent manner in response to pretreatment (3–24 h) of 0.4 M NaCl peptide (5 µg/mL) as indicated in Fig. 3a. Figure 3b presents the time-dependent increment of %apoptosis in H460 cells preincubated with 0.4 M NaCl peptide. Correspondingly, nuclear costaining with Hoechst33342 and propidium iodide (PI) also showed that apoptosis cell death was remarkably noted in *L. squarrosulus* peptide-pretreated H460 cells in a time (6–24 h) -dependent manner (Fig. 3c).

Flow cytometry with annexin V-FITC/PI was performed to further clarify mode of cell death and evaluate the sensitizing effect of *L. squarrosulus* peptide on cisplatin-induced apoptosis in human lung cancer H460 cells. Labelled annexin V-FITC allows the accurate tracking of the early apoptosis cells due to specific binding to the exposed phosphatidylserine on the surface of cell membrane^34^. Annexin V-FITC is used in conjugation with PI to further track the cells undergoing late apoptosis and necrosis, cell integrity is compromised thereby allowing PI internalization^35^. The obtained histograms indicated that majority of the cisplatin-treated and peptide-treated H460 were viable cells, comparable to the untreated cell population, as indicated by the large accumulation of non-stained cells (Annexin V-/PI-) in the three groups (Fig. 3d). On the other hand, there was remarkable increase in the percentage of cells in both early (Annexin V + /PI-) and late (Annexin V + /PI +) apoptosis in human lung cancer cells pretreated with 5 µg/mL of 0.4 M NaCl peptide for 6–24 h following with further 24 h treatment of 5 µM cisplatin (Fig. 3e). These results indicate that the *L. squarrosulus* peptide augments cisplatin-induced apoptosis when non-toxic concentrations were used for both cisplatin and the peptide, thus providing further evidence of a strong chemosensitization effect of *L. squarrosulus* peptide.

### Diminution of colony formation in cisplatin-treated H460 cells precultured with *L. squarrosulus* peptide

The ability of lung cancer cells to form new colonies after cisplatin treatment alone or with 0.4 M NaCl fraction from *L. squarrosulus* extracts was investigated through clonogenic assay, which is an in vitro assay often used to gauge the effect of a treatment on cell survival and proliferation^36^. Viable H460 cells were collected after preincubation with 0–5 µg/mL of 0.4 M NaCl peptide for 24 h following with the further incubation of 5 µM cisplatin for another 24 h. A single cell suspension was prepared and seeded onto 6-well plate at a density of 250 cells/well, and the culture was maintained for 7 days. Figure 3f depicts that in comparison with *L. squarrosulus* peptide-treated H460 cells and the untreated control group which presented evidently higher colony number, colony formation was diminished in cisplatin-treated cells. Remarkably, the reduction of colony formation was greater in cisplatin-treated H460 cells which were preincubated with *L. squarrosulus* peptide (Fig. 3g).

### *L. squarrosulus* peptide modulates apoptosis and integrin-mediating survival pathway

**Figure 4:**
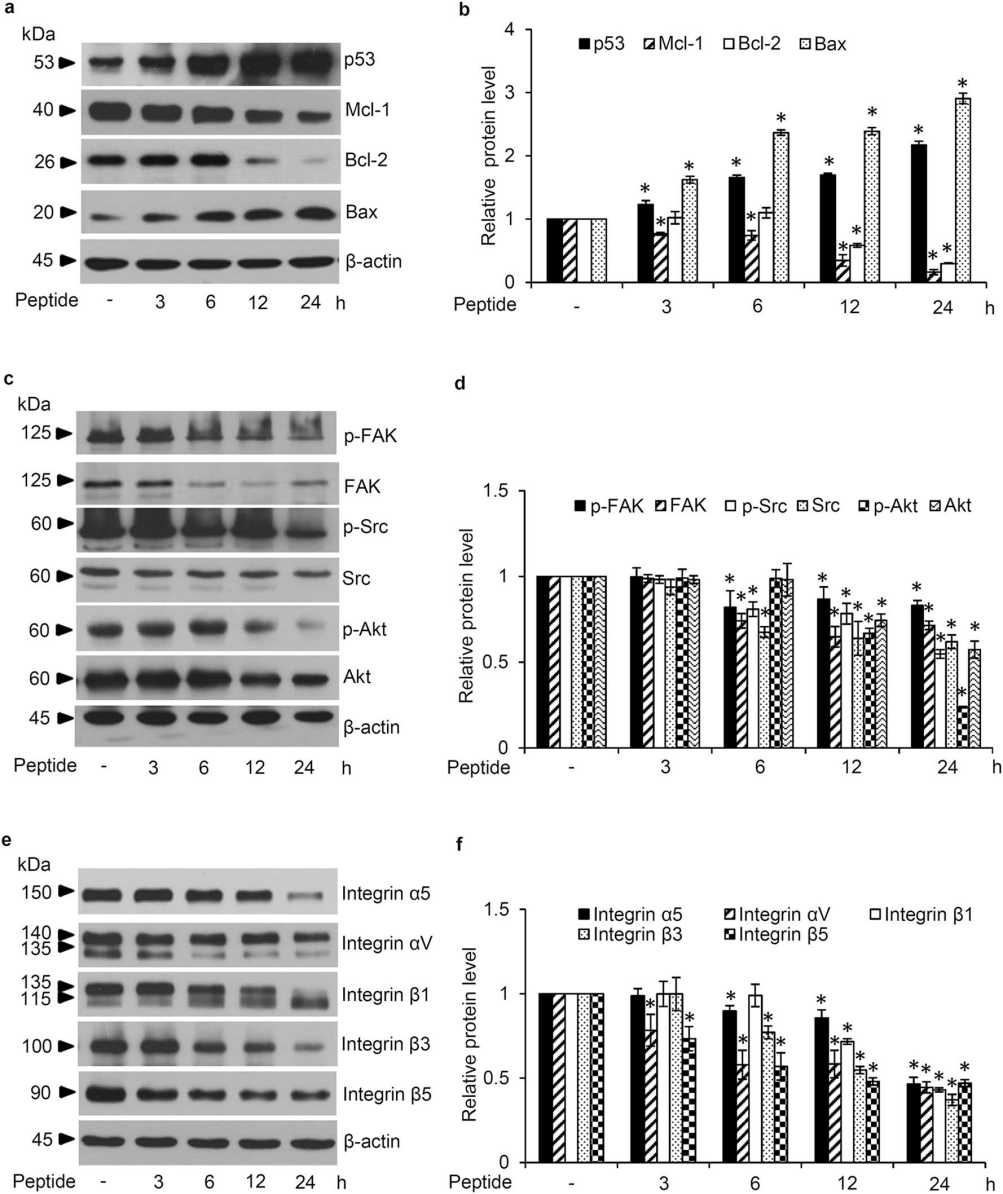
*L. squarrosulus* peptide modulates apoptosis and survival pathway in human lung cancer cells. (**a**) Western blot analysis revealed the reduction of anti-apoptosis proteins (Mcl-1, Bcl-2) in H460 cells cultured with 5 µg/mL of *L. squarrosulus* peptide from 0.4 M NaCl fraction for 12–24 h. (**b**) However, the significant upregulation of p53 and pro-apoptosis Bax protein was observed after 3 h of peptide treatment. The downregulation of (**c**) survival proteins and (**e**) upstream regulatory molecules, integrins, was also detected in *L. squarrosulus* peptide treated-cells. (**d**) The expression level of survival p-FAK, FAK, p-Src, Src, p-Akt and Akt protein was time-dependently decreased which corresponded with (f) the diminution of integrin β1, β3, β5, αV and α5 in H460 cells incubated with 5 µg/mL of 0.4 M NaCl peptide. Values are means of three independent experiments ± SD. *p < 0.05 versus non-treated control cells.

The alteration on apoptosis-regulating proteins was examined in human lung cancer cells cultured with *L. squarrosulus* peptide for varying time periods (0–24 h). Augmentation of tumor suppressor p53 and pro-apoptosis Bax proteins as well as downregulation of anti-apoptosis Mcl-1 protein were time-dependently observed in H460 cells incubated with 0.4 M NaCl peptide at 5 µg/mL for 3–24 h (Fig. 4a). The alteration of these apoptosis-regulating proteins correlated well with the chemosensitizing activity of *L. squarrosulus* peptide. Moreover, the expression of Bcl-2 anti-apoptosis protein was significantly decreased in lung cancer cells after treatment with the peptide for 12–24 h compared with non-treated control cells (Fig. 4b).

The evaluation on modulatory role of *L. squarrosulus* peptide on survival signaling pathways in human lung cancer cells was further performed. Western blot analysis revealed incrementally decreased expression of survival-related proteins including FAK, p-FAK, Src, p-Src, Akt, p-Akt in H460 cells cultured with 0.4 M NaCl peptide at 5 µg/mL for 6–24 h (Fig. 4c,d). Moreover, the diminished levels of β1, β3, β5, α5 and αV integrins, which are up-stream regulatory molecules, were time-dependently observed following the treatment of *L. squarrosulus* peptide (Fig. 4e,f). These results indicated that *L. squarrosulus* peptide at non-toxic concentration effectively alters apoptosis and integrin-mediating survival signals in human lung cancer cells.

### Anticancer mechanisms of cisplatin promoted by *L. squarrosulus* peptide

**Figure 5:**
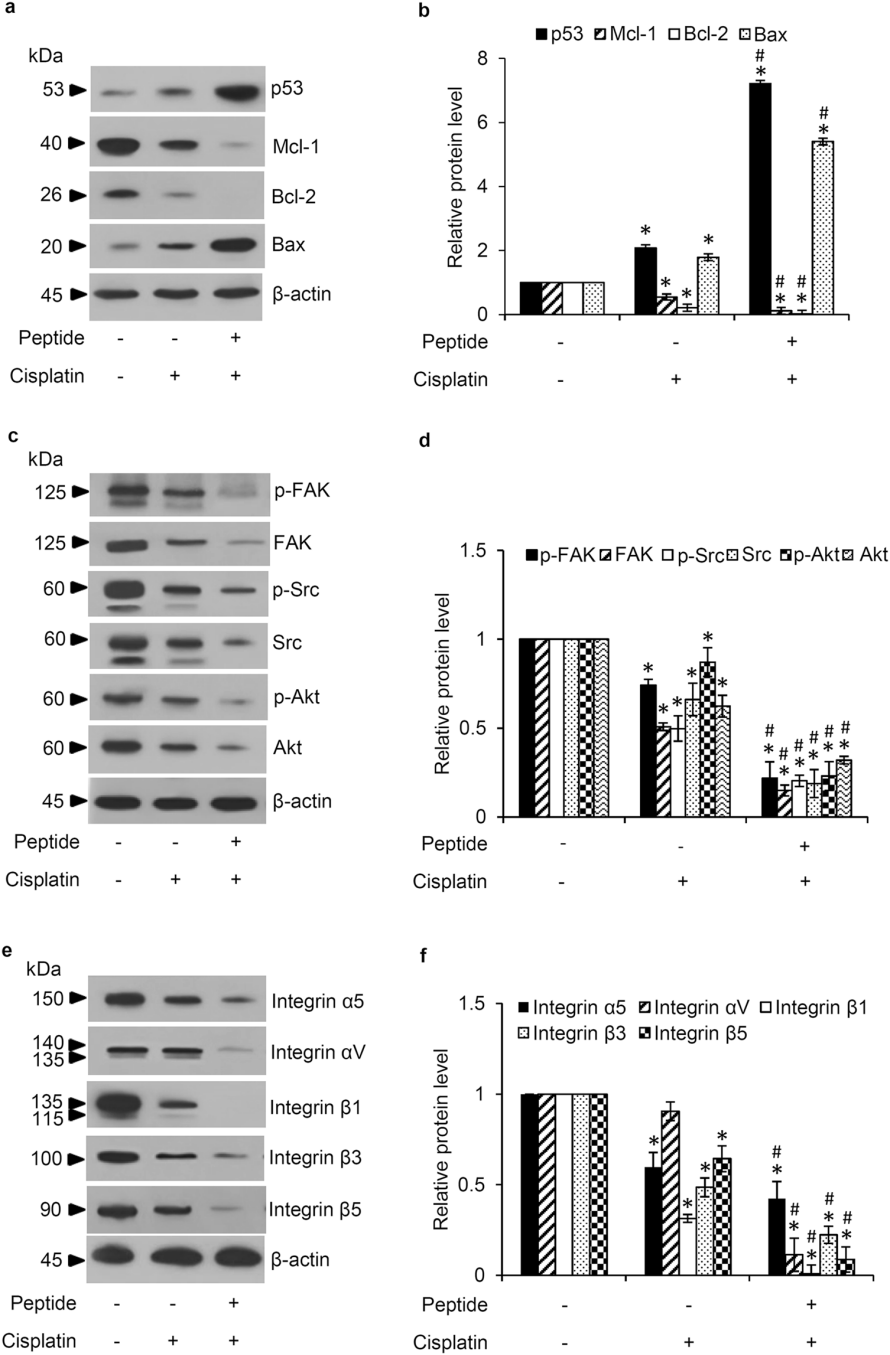
Pretreatment with 0.4 M NaCl peptide obtained from *L. squarrosulus* significantly sensitizes cisplatin-induced cell death through regulation of apoptosis and integrin-mediated survival pathway in human lung cancer H460 cells. (**a**, **b**) The protein levels of Mcl-1 and Bcl-2 were significantly decreased while tumor suppressor p53 and pro-apoptosis Bax protein levels were obviously increased in comparison with H460 cells treated with cisplatin alone. (**c**, **d**) Related survival proteins (FAK, p-FAK, Src, p-Src, Akt, p-Akt) and (e, f) integrins (α5, αV, β1, β3, β5) were considerably decreased as a result of peptide preincubation in cisplatin-treated cells compared to the cells treated with cisplatin alone. Values are means of three independent experiments ± SD. *p < 0.05 versus non-treated control cells, ^#^*p* < 0.05 versus the cells only treated with cisplatin.

The results in Fig. 4 show that incubation with *L. squarrosulus* peptide alone modulated the expression levels of signaling proteins involved in apoptosis and survival pathways in human lung cancer cells. These proteins were likewise investigated in *L. squarrosulus* peptide-preincubated H460 cells which received cisplatin treatment. Culture with 5 µM of cisplatin alone for 24 h significantly altered the level of Bcl-2 family proteins including Bax, Mcl-1 and Bcl-2 showing that intrinsic apoptosis pathway is activated in response to cisplatin treatment (Fig. 5a). The upregulation of p53 and Bax as well as the reduction of Mcl-1 and Bcl-2 were significantly greater in the 0.4 M NaCl peptide-pretreated H460 cells subjected to cisplatin treatment in comparison to the cisplatin-treated cells (Fig. 5b). Similarly, the suppression on integrin-mediating survival pathway was also indicated in cisplatin-treated H460 cells. The decreased levels of integrin β1, β3, β5 and α5 and the corresponding diminution of survival signaling proteins including FAK, p-FAK, Src, p-Src, Akt and p-Akt were more pronounced in the cisplatin treated-H460 cells that were precultured with 0.4 M NaCl peptide compared with the cells treated with only cisplatin (Fig. 5c,d,e,f). Intriguingly, the downregulation of integrin αV in human lung cancer cells was only observed in response to pretreatment with *L. squarrosulus* peptide followed by the exposure to cisplatin.

### Selective chemosensitizing effect of *L. squarrosulus* peptide in human lung cancer cells

**Figure 6:**
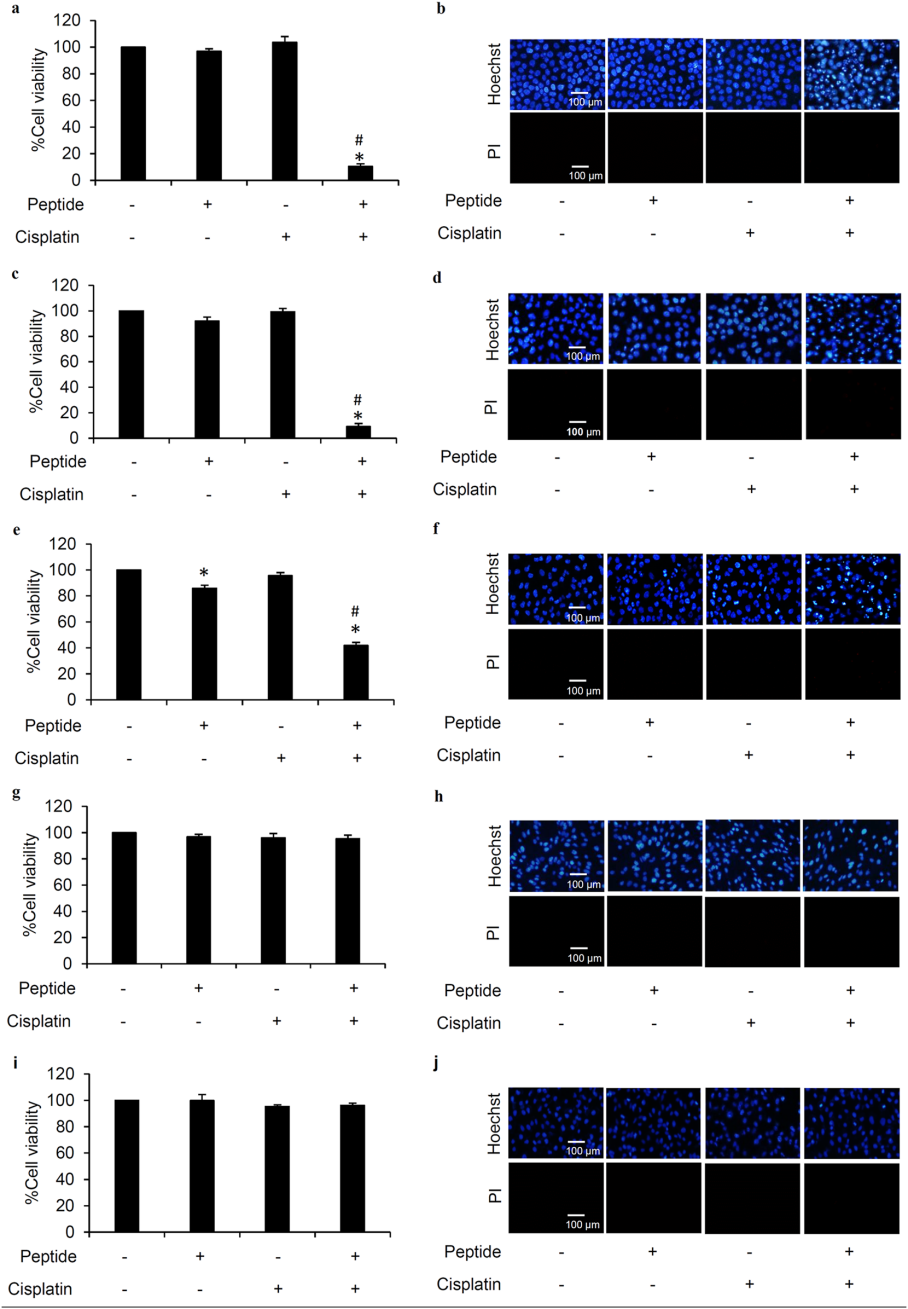
Peptide from *Lentinus squarrosulus* Mont. shows high safety prolife. Chemosensitizing effect of 0.4 M NaCl peptide from *L. squarrosulus* was presented in various human lung cancer cells including (**a**) H23, (**c**) H292 and (**e**) A549 cells. In comparison to cells with or without cisplatin treatment, peptide preincubation combined with cisplatin treatment augmented observed apoptosis cells which are characterized by condensed DNA/fragmented nuclei stained by bright blue fluorescence of Hoechst33342 in (**b**) H23, (**d**) H292 and (**f**) A549 cells. MTT assay revealed no alteration of cell viability in (**g**) human dermal papilla DPCs cells and (**i**) human proximal renal HK-2 cells after preculture with 5 µg/mL of 0.4 M NaCl peptide for 24 h following with treatment with 5 µM cisplatin for another 24 h. No detectable apoptosis and necrosis were noted via costaining with Hoechst33342/propidium iodide (PI) in all treatments of (**h**) DPCs and (**j**) HK-2 cells. Values are means of three independent experiments ± SD. *p < 0.05 versus non-treated control cells, ^#^*p* < 0.05 versus the cells treated only with cisplatin.

To further assess the chemosensitizing effect previously observed in H460 (p53 and KRas wild-type) (Fig. 3), other human lung cancer cells including H292 (p53 wild-type), H23 (p53 and KRas mutant) and A549 (KRas mutant) were either pretreated with 5 µg/mL of 0.4 M NaCl peptide for 24 h or left untreated before further incubation with non-toxic concentration of cisplatin at 5 µM for 24 h. Figure 6a,c,e, respectively revealed the greater reduction of %cell viability in cisplatin-treated H23, H292 and A549 lung cancer cells which were preincubated with *L. squarrosulus* peptide compared with the cells treated with cisplatin alone. While there were no observable apoptosis and necrosis cells detected after nuclear staining with Hoechst33342 and PI in lung cancer cells cultured with cisplatin or 0.4 M NaCl peptide alone for 24 h, apoptosis was strongly augmented in cisplatin-treated cells that were preincubated with *L. squarrosulus* peptide (Fig. 6b,d,f).

Due to notable cisplatin-modulated serious adverse effects such as hair loss and nephrotoxicity^30–32^, the effect of *L. squarrosulus* peptide on cisplatin-induced toxicity was also evaluated in human dermal papilla DPCs and proximal renal HK-2 cells. Figure 6g,h revealed that 24 h pretreatment with 0.4 M NaCl peptide at 5 µg/mL did not cause significant alteration in viability and death in DPCs incubated with cisplatin (5 µM) for 24 h when compared with untreated control and cells with cisplatin treatment alone. Furthermore, there was no cisplatin-induced toxicity as reflected by the MTT viability assay and Hoechst33342/PI costaining performed in HK-2 cells precultured with *L. squarrosulus* peptide prior exposure to cisplatin (Fig. 6i,j). These results suggested that *L. squarrosulus* peptide selectively sensitized cisplatin toxicity in human lung cancer cells**.**

**Figure 7:**
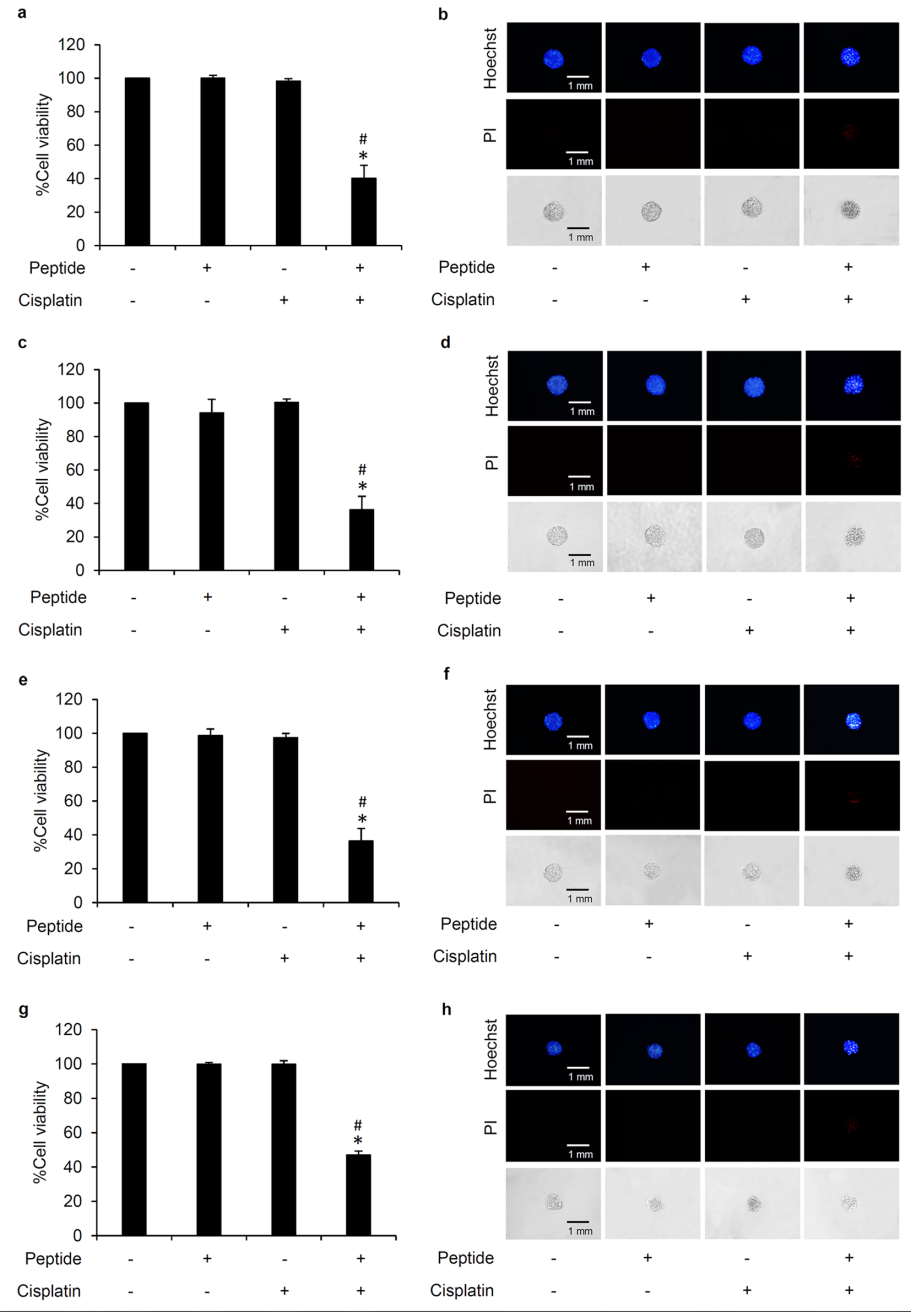
Chemosensitizing effect of *L. squarrosulus* peptide in multicellular lung cancer spheroids. PrestoBlue assay presented the significant reduction of %cell viability in cancer spheroids obtained from lung cancer (**a**) H460, (**c**) H23, (e) H292 and (**g**) A549 cells that were cultured with 0.4 M NaCl peptide (5 µg/mL) for 24 h prior to 24 h treatment of 5 µM cisplatin. Nuclear staining clearly depicted the elevation of apoptosis observed as bright blue fluorescence of Hoechst33342 in multicellular spheroids of (**b**) H460, (**d**) H23, (**f**) H292 and (**h**) A549 cells after coculture with *L. squarrosulus* peptide and cisplatin. Notably, the presentation of both bright blue Hoechst33342 fluorescence and red fluorescence of propidium iodide (PI) indicated late stage of apoptosis detected in the spheroids after preincubation with 0.4 M NaCl peptide followed by cisplatin treatment. Values are means of three independent experiments ± SD. *p < 0.05 versus non-treated control cells, ^#^*p* < 0.05 versus the cells treated only with cisplatin.

Additionally, chemosensitizing effect of the peptide from *L. squarrosulus* was validated in three-dimensional cancer spheroids. The in vitro multicellular cancer spheroids better reflect in vivo tumor behavior and thus the spheroid culture has been recognized as a good model for evaluation of anticancer activity^37,38^. Similar to the effects seen in two-dimension cell culture, preincubation with 0.4 M NaCl peptide at 5 µg/mL for 24 h prior to treatment with cisplatin remarkably diminished %cell viability of cancer spheroids derived from lung cancer H460 (Fig. 7a), H23 (Fig. 7c), H292 (Fig. 7e) and A549 (Fig. 7g) cells. Meanwhile, culture alone either with 0.4 M NaCl peptide (5 µg/mL) or cisplatin (5 µM) for 24 h did not alter viability in all lung cancer spheroids. Nuclear staining obviously depicted apoptosis cell death evidenced by the bright blue fluorescence of Hoechst33342 in the spheroids treated with *L. squarrosulus* peptide following with cisplatin treatment (Fig. 7b,d,f,h). These results strongly support the chemosensitizing effect of *L. squarrosulus* peptide on cisplatin-induced apoptosis in human lung cancer cells.

## Discussion

Cisplatin is one of the most clinically effective anticancer agents and it remains to be a component of first-line treatment for advanced non-small cell lung cancer^39^. However, drug resistance and numerous undesirable side effects limit the use of cisplatin. To combat these problems and obtain greater therapeutic outcome, the combination of cisplatin with other drugs has been an available option^40^. Chemosensitization by natural compounds to increase cisplatin potency and decrease substantial toxicity is gaining interest as a novel anticancer strategy^27^. Thus, this study evaluated the chemosensitizing effect of the peptide extracted from *L. squarrosulus*, an edible mushroom, on cisplatin-induced cell death in human lung cancer cells. Time-dependent chemosensitizing activity was evidenced by the augmentation of cisplatin-induced apoptosis detected with Hoechst33342/PI costaining (Fig. 3b,c) and annexin V-FITC/PI assay through flow cytometry (Fig. 3d,e) of human lung cancer cells pretreated with 5 µg/ml of purified 0.4 M NaCl peptide from *L. squarrosulus* extracts for 6–24 h.

Cisplatin cytotoxicity is mediated by the propagation signals produced in response to DNA damage which in turn activate downstream proteins such as p53 and pro-apoptosis Bax, ultimately resulting in apoptosis^40,41^. The dysregulation of apoptosis, especially through the intrinsic pathway controlled by the Bcl-2 family proteins, could contribute to the resistance to cisplatin-induced toxicity observed in cancer cells^41^. Indeed, the elevated level of anti-apoptosis proteins in the Bcl-2 family has been well recognized as an underlying mechanism of chemotherapeutic resistance^42^. The downregulation of p53 and Bax in lung cancer cells is also associated with increased cisplatin resistance and reduced apoptosis^10^. Moreover, tumor progression is tightly correlated with decreased level of tumor suppressor p53 protein^43^. The treatment with *L. squarrosulus* peptide alone in human lung cancer cells caused not only the reduction of anti-apoptosis Mcl-1 and Bcl-2 proteins but also the augmentation of p53 and Bax in a time-dependent manner (Fig. 4a). Besides that, pretreatment with the peptide dramatically enhanced the effects of cisplatin treatment at non-toxic concentration, such as reduction of Mcl-1 and Bcl-2 levels as well as the elevation of p53 and Bax in cisplatin-treated lung cancer cells (Fig. 5a).

Integrins are heterodimeric transmembrane adhesion proteins which serve as survival initiating molecules. Cellular signaling generated from the interaction between ECM and integrins activates down-stream survival proteins including FAK and Src which subsequently propagates the PI3K/Akt pathway and its strong survival-related effects^18^. Downregulation of integrins (β1, β3, β5, α5 and αV) corresponds to the suppression of activated forms of survival signaling molecules, p-FAK, p-Src and p-Akt in lung cancer cells cultured with *L. squarrosulus* peptide (Fig. 4c-f). Interestingly, it has been continuously reported that various natural peptides could effectively repress the integrin-regulated survival pathways^44,45^. Integrins, especially the β1, β3, β5, α5 and αV subunit, and the related survival proteins have been shown to contribute to uncontrollable lung cancer progression and chemotherapeutic failure, as their dysregulation could promote rapid cloning of cancer cells and increase cell survival against various stresses such as cisplatin toxicity^19,20,46^. Coinciding with decreased survival signaling, the capability of individual cells to generate new cancer colonies was clearly more suppressed in cisplatin-treated lung cancer cells which were then preincubated with *L. squarrosulus* peptide as compared to both untreated control and cisplatin-treated groups (Fig. 3e,g). This inhibitory activity of *L. squarrosulus* peptide on colony formation correlated well with observed lower level of integrin-regulated survival proteins in lung cancer cells preincubated with *L. squarrosulus* following with cisplatin treatment in comparison with cells cultured only with cisplatin (Fig. 5c-f).

Integrin signaling allows the survival of lung cancer cells against drug-induced apoptosis. Thus, targeting of integrins presents good therapeutic potential for lung cancer cells treatment^24,46^. Downregulation of integrins and associated survival signals induces apoptosis and suppresses survival in diverse lung cancer cell, as observed in both p53 wild-type and mutants lung cancer cells^46,47^. Moreover, activation of integrins (β1, β3, αV) is also associated with aggressive features of oncogenic KRas dependent lung cancer cells and antagonizing these integrins has been shown to sensitize the resistant lung cancer cells to chemotherapy^48–50^. The presented results indicate that *L. squarrosulus* peptide may provide chemosensitizing activity against wild-type and mutant lung cancer cells through inhibition of integrins as demonstrated by the peptide’s effects on H460 (p53 and KRas wild-type), H292 (p53 wild-type), H23 (p53 and KRas mutant) and A549 (KRas mutant) (Figs. 3, 6, 7).

In line with the results presented in this study, there is much evidence which suggest that integrin is a likely target molecule affected by anticancer peptides^44,45,51^. The disruption on integrin-ECM interaction can trigger the internalization and degradation of transmembrane integrins^52,53^. Although downregulated level of integrins and consequent repression of downstream survival signals support that integrin is a potential therapeutic target of *L. squarrosulus* peptide, other related mechanisms of anticancer peptides such as perturbation of cell membrane and penetration to intracellular organelles are worthy of further elucidation^54^.

Not only selective anticancer activity against human lung cancer cells (Fig. 2) but also specific chemosensitizing effect of the obtained *L. squarrosulus* peptide was clearly evident as the results indicate that the enhancement of cisplatin-induced apoptosis occurred only in human lung cancer cells but not in non-cancer DPCs and HK-2 cells (Fig. 6). These results suggest that the *L. squarrosulus* peptide may provide protective effect against cisplatin side effects such as hair loss and nephrotoxicity which correlate with increased cell death in dermal papilla and proximal renal cells, respectively^31,32^. The selective chemo-enhancing activity against human lung cancer cells of *L. squarrosulus* peptide would thus be beneficial in reducing the dose-limiting cisplatin toxicity observed in normal cells.

Although initial assessment suggests the eluted 0.4 M NaCl fraction is composed of a ~ 37 kDa peptide (Fig. 1), the proteomics analysis revealed many amino acid sequences that did not match with available peptides in the library. Further improvement in purification process might facilitate the identification of the novel chemosensitizing peptide. However, the highest recorded similarity score in the proteomics analysis corresponded to extracellular metalloproteinase *Ganoderma sinense* and *Lentinus tigrinus* (Fig. 1c), confirming the mushroom origins of this chemotherapeutic adjuvant peptide and encouraging greater attention to these natural sources.

**Figure 8:**
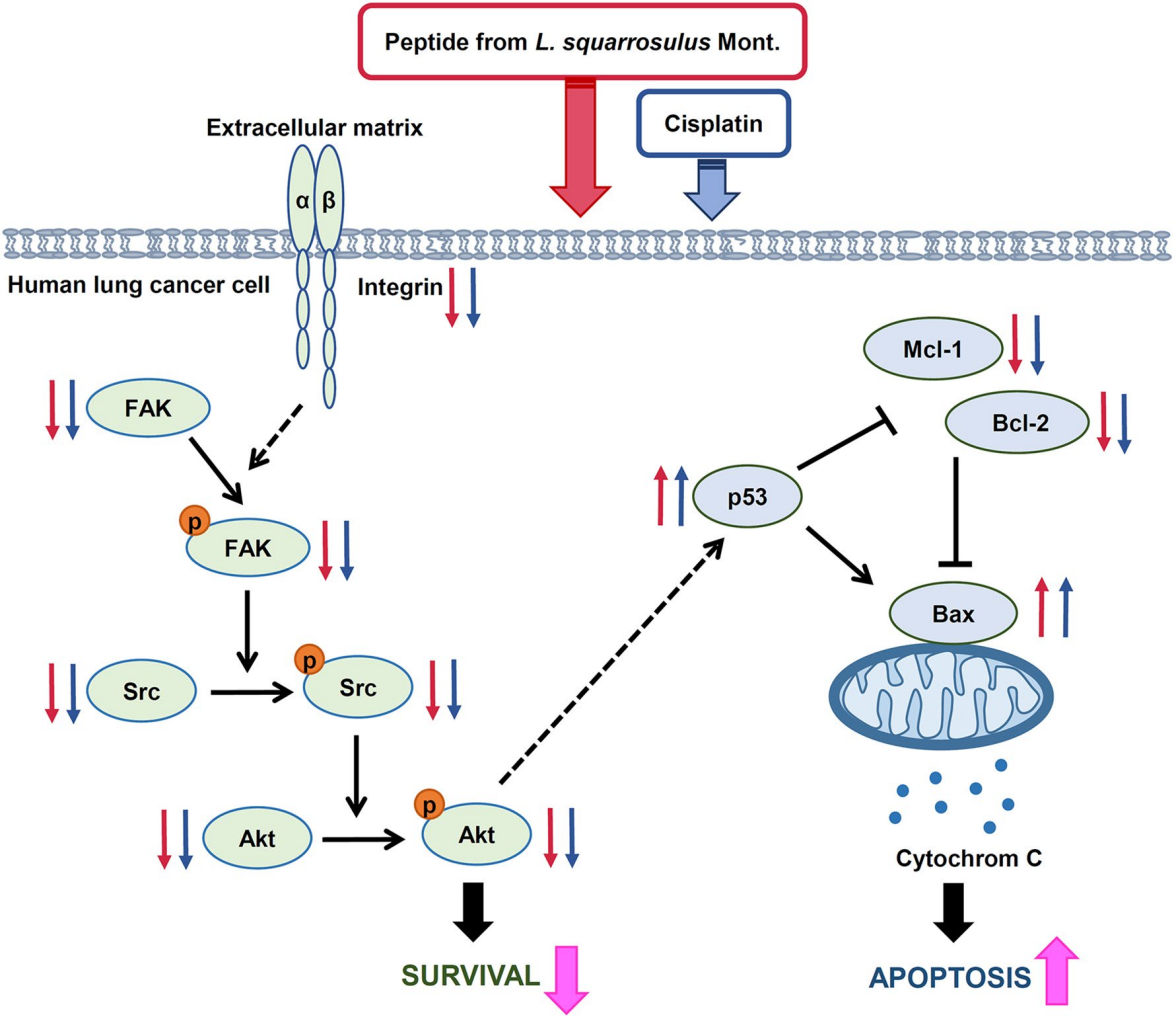
Proposed regulatory machinery of chemosensitizing effect of peptide extracted from *Lentinus squarrosulus* on cisplatin-induced apoptosis in human lung cancer cells. The peptide enhances the suppressive activity of cisplatin on integrin-mediating survival which resultes in the downregulation of integrin and down-stream signaling proteins including p-FAK/FAK, p-Src/Src and p-Akt/Akt as well as activation of apoptosis mediated by overexpression of p53 which correlates with increased Bax and diminution of anti-apoptosis Mcl-1 and Bcl-2 proteins. The effects of the peptide and cisplatin at non-toxic concentrations is indicated by red and blue small arrows, respectively. Big pink arrows represent the augmented cytotoxicity of cisplatin after pretreatment of human lung cancer cells with 0.4 M NaCl fraction of *L. squarrosulus* peptides.

In summary, the current study revealed selective chemosensitizing effect of *L. squarrosulus* peptide extract to cisplatin-induced apoptosis in human lung cancer cells via suppression of integrin/FAK/Src/Akt survival signaling, downregulation of anti-apoptosis proteins (Mcl-1, Bcl-2), and upregulation of p53 as well as pro-apoptosis Bax proteins (Fig. 8). Subsequently, the data presented herein would support the development of *L. squarrosulus* peptides as a novel chemosensitizer.

## Methods

### Chemical reagents

Hoechst33342, propidium iodide (PI), dimethylsulfoxide (DMSO), cisplatin, 3-(4,5-dimethylthiazol-2-yl)-2,5-diphenyltetrazolium bromide (MTT), Tween-20, acetonitrile, formic acid, coomassie brilliant blue R-250, isopropanol, acetic acid and skim milk powder were bought from Sigma-Aldrich Chemical (St. Louis, MO, USA). Annexin V-Fluorescein Isothiocyanate (FITC) apoptosis detection kit and PrestoBlue reagent were procured from Thermo Fisher Scientific (Rockford, IL, USA). Roswell Park Memorial Institute (RPMI) medium, Dulbecco’s Modified Eagle Medium (DMEM), fetal bovine serum (FBS), l-glutamine, penicillin/streptomycin solution and trypsin–EDTA (0.25%) were purchased from Gibco (Gaithersburg, MA, USA). Ammonium sulfate ((NH_4_)_2_SO_4_), potassium dihydrogen phosphate (KH_2_PO_4_), ammonium bicarbonate (NH_4_HCO_3_), dithiothreitol, iodoacetamide, methanol (CH_3_OH) and ethanol (CH_3_CH_2_OH) were obtained from Merck (Darmstadt, Germany). Di-sodium hydrogen orthophosphate anhydrous (Na_2_HPO_4_), potassium chloride (KCl) and sodium chloride (NaCl) were obtained from Univar (Ajax, Australia). Primary antibodies specific for integrins α5, αV, β1, β3, and β5, FAK, p-FAK (Tyr 397), Src, p-Src (Tyr 416), Akt, p-Akt (Thr 308), p53, Bax, Mcl-1, Bcl-2, β-actin as well as the horseradish peroxidase (HRP)-labeled secondary antibodies were purchased from Cell Signaling Technology (Danvers, MA, USA). Immobilon Western chemiluminescent HRP substrate was obtained from Milipore (Billerica, MA, USA). A bicinchoninic acid (BCA) protein assay kit was purchased from Thermo scientific (Rockford, IL, USA).

### Preparation of extracted peptide

- Isolation of crude peptides
  The edible mushrooms, *L. squarrosulus*, were homogenized with deionized water (3 mL/g). Proteins were salted out slowly on ice by adding (NH_4_)_2_SO_4_ to the aqueous filtrate until 40–80% final saturation. Next, centrifugation at 6,500 rpm for 1 h (4 °C) was performed to acquire crude protein pellets which were re-solubilized with phosphate buffer solution (PBS; pH 7.4) containing Na_2_HPO_4_ and KH_2_PO_4_ on ice. Then, the solution was dialyzed overnight with the phosphate buffer at 4 °C to eliminate (NH_4_)_2_SO_4_ as previously described^29^.
- Peptide purification
  Fast protein liquid chromatography (FPLC) with HiTrap DEAE FF 1 mL anion exchange column pre-equilibrated with PBS (pH 7.4) was used for further peptide purification. After elution of the unbound peptides, the different adsorbed peptides were released from the positively charged adsorbent via stepwise increase of NaCl concentration (0.1, 0.2, 0.3, 0.4 and 0.5 M) in PBS at a flow rate of 1 mL/min. The eluted peptides were further concentrated via lyophilization. The eluates were then analyzed for quantity and homogeneity of peptides by BCA assay kit and SDS-PAGE (sodium dodecyl sulfate polyacrylamide gel electrophoresis), respectively.
- Determination of protein content
  BCA assay kit was used to measure the total protein content of each concentrated peptide extract, according to manufacturer’s instructions. The developed product was evaluated through microplate reader (Anthros, Durham, NC, USA) at 570 nm. The protein concentration was calculated based on the calibration curve of the BSA standard.
- Evaluation on homogeneity of purified peptide fraction
  The homogeneity of purified peptide fraction was determined by SDS-PAGE using 15% (w/v) gel as previously described^29^. Briefly, the mixtures containing 15 µg protein content of each peptide fraction and loading dye were added onto SDS-PAGE after heating at 95 °C for 5 min. After complete separation, the gel was stained with coomassie brilliant blue R-250 solution overnight and then destained with isopropanol: acetic acid: water (10: 10: 80% v/v) solution.
- Identification of peptide in purified fraction from *L. squarrosulus* extracts

The most purified fraction from *L. squarrosulus* extracts presenting only one major protein band via SDS-PAGE analysis was selected for further peptide identification. The SDS-PAGE gel area of interested protein was cut and washed with sterile deionized water. After destaining with 25 mM NH_4_HCO_3_ in 50% methanol, the gel was washed three times with sterile deionized water. Before incubation with 10 mM dithiothreitol (in 10 mM NH_4_HCO_3_) at room temperature for 1 h, the gel was dehydrated by soaking in acetonitrile. After adding 5 mM dithiothreitol (in 50 mM iodoacetamide), the gel was subsequently incubated for 1 h in the dark. Then, the gel was washed with acetonitrile (2 time × 5 min) and digested with 10 ng/μL of proteomics-grade trypsin (Sigma-Aldrich Chemical, St. Louis, MO, USA) in 50% acetonitrile/10 mM NH_4_HCO_3_ at 37 °C for 12 h. The supernatant was collected and concentrated through vacuum-drying. The peptide sample was kept at - 80 °C until performing proteomics analysis. Before the sample was subjected to mass spectrum analysis, it was solubilized in 0.1% formic acid. LC–MS/MS with nanocolumn was set at a flow rate of 300 nL/min using the HCT Ultra PTM Discovery System (Bruker Daltonics Ltd., Hamburg, Germany) coupled to an UltiMate 3000 LC System (Dionex Ltd., Sunnyvale, CA, USA). A multistep gradient of 10–70% acetonitrile (in 0.1% formic acid) was used as mobile phase to elute peptides^55^. The mass spectrum fingerprint of the eluted peptides was searched against Contaminants database version 20090624 (262 sequences, 133770 residues) and NCBIprot database version 20191120 (227181163 sequences; 82759882099 residues) using Mascot search web service (Matrix Science, London, UK) with the following search parameters; Type of search: MS/MS Ion Search, Enzyme: Trypsin, Fixed modifications: Carbamidomethyl (C), Variable modifications: Oxidation (M), Mass values: Monoisotopic, Protein Mass: Unrestricted, Peptide Mass Tolerance: ± 1.2 Da, Fragment Mass Tolerance: ± 0.6 Da, Max Missed Cleavages: 1, Instrument type: ESI-TRAPT. Protein hits was considered positively identified when a component peptide had a statistically significant Mascot score (*p* < 0.05). The matched peptide sequences were searched against UniProtKB reference proteomes plus Swiss-Prot database in UniProt release 2020_03 for protein annotation.

### Cell culture

Human lung cancer H460, H23 and H292 cells (ATCC, Manassas, VA, USA) were cultured in RPMI medium. Meanwhile, human lung cancer A549 cells (ATCC, Manassas, VA, USA) and human renal proximal tubular HK-2 cells (ATCC, Manassas, VA, USA) were cultured in DMEM. Prigrow III medium (Applied Biological Materials Inc., Richmond, BC, Canada) was used for the culture of human dermal papilla cells DPCs obtained from Applied Biological Materials Inc. (Richmond, BC, Canada). All culture mediums were supplemented with l-glutamine (2 mM), FBS (10%) and penicillin/streptomycin (100 units/mL). The cells were maintained in humidified atmosphere containing 5% CO_2_ with temperature set at 37 °C until 70–80% confluence for further experiments.

### Cytotoxicity assay

Cells were seeded onto 96-well plate at a density of 1 × 10^4^ cells/well for overnight. After indicated treatment, the culture medium was replaced with 0.4 mg/mL of MTT and the cells were further incubated for 3 h at 37 °C in dark place. Then, the supernatant was removed and DMSO was added to dissolve the formazan product. A microplate reader (Anthros, Durham, NC, USA) was used to measure the intensity of formazan color at 570 nm. The absorbance ratio of treated to non-treated control cells was calculated and presented as percent cell viability.

### Nuclear staining assay

Mode of cell death was evaluated via nuclear staining assay. The treated cells were costained with Hoechst33342 (0.02 µg/mL) and PI (0.01 µg/mL) at 37 °C for 30 min. A fluorescence microscope (Olympus IX51 with DP70, Olympus, Tokyo, Japan) was used to visualize the cells and characterize the mode of death. The Hoechst33342 dye was allowed for the detection of nuclear condensation which characterizes apoptosis while the necrosis cells were distinguished by their uptake of PI.

### Flow cytometric analysis

The percentages of cells undergoing apoptosis and necrosis were also quantified through flow cytometry after double staining using annexin V-FITC/PI assay kit, following manufacturer’s instructions. Briefly, lung cancer H460 cells were cultured at the density of 1.5 × 10^5^ cells/well in 6-well plate. The cells were preincubated with the peptide extract for varying times (0, 6, 12, 24 h) before further culture with 5 µM of cisplatin for 24 h. Then, the cells were detached and centrifuged at 5,000 rpm (4 °C) for 5 min. The annexin V-FITC and PI working solution were added into respective single cell suspensions which were prepared in binding buffer solution. The proportions of living, apoptosis and necrosis cells in each prepared suspension were determined with Guava easyCyte 5 benchtop flow cytometer with the GuavaSoft version 2.7 software (Merck, Darmstadt, Germany).

### Clonogenic assay

Human lung cancer cells (1.5 × 10^5^ cells/well) in 6-well plate were pretreated with peptide (5 µg/mL) for 24 h before exposure with 5 µM of cisplatin. After 24 h, single cell suspensions of 250 viable cells derived from each treatment were seeded again onto 6-well plate. The colony formation in each plate was examined after 7 days of incubation under 5% CO_2_ humidified atmosphere at 37 °C. The resulting cancer colonies were counted after staining with 0.05% w/v crystal violet in 4% formaldehyde^36^.

### Establishment of three-dimensional multicellular cancer spheroids

Human lung cancer cells were seeded at a density of 1 × 10^4^ cells/well and maintained with appropriate media containing 10% FBS, 2 mM l-glutamine and 100 units/mL penicillin/streptomycin in 96-well round bottom ultra-low attachment plate (Corning, Tewksbury, MA, USA). After 4-day incubation, the lung cancer spheroids were subjected to the evaluation of chemosensitizing activity. To examine viability of lung cancer cells, the three-dimensional spheroids were incubated with PrestoBlue reagent (10 µL) for 3 h. Then, the fluorescence intensity of resorufin, the reduced form of resazurin was detected with fluorescence microplate reader (CLARIOstar Plus Microplate Reader, BMG Labtech, Baden-Wurttemberg, Germany) at excitation wavelength of 570 nm and emission wavelength of 610 nm. The percent cell viability was derived from the relative fluorescence intensity between treated to non-treated spheroids. Moreover, mode of cell death was observed under fluorescence microscope (Olympus IX51 with DP70, Olympus, Tokyo, Japan) after costaining tumor spheroids with Hoechst33342 (0.02 µg/mL) and PI (0.01 µg/mL) at 37 °C for 30 min^56^.

### Western blot analysis

Human lung cancer H460 cells were seeded onto 6-well plate at the density of 1.5 × 10^5^ cells/well. After indicated treatments, the cells were incubated with lysis buffer containing 1X radio-immunoprecipitation assay (RIPA) buffer (Thermo scientific, Rockford, IL, USA) and cocktail protease inhibitor (Roche Applied Science, Indianapolis, IN, USA) for 45 min on ice. To collect the clear supernatant, the cell lysates were centrifuged at 10,000 rpm at 4 °C for 15 min. BCA protein assay kit was used to determine total protein content. Equal amount of protein from each sample was adjusted to appropriate volume using lysis buffer and then mixed with loading buffer. The samples were heated at 95 °C for 5 min to speed up protein denaturation. Then, 35 µg protein from each sample was loaded onto a 10% SDS-PAGE. After separation, the cellular proteins were transferred onto 0.45 µm nitrocellulose membranes (Bio-Rad Laboratories, Hercules, CA, USA). The membranes were blocked with 5% non-fat dry milk in Tris buffered saline with Tween 20 (TBST) at room temperature for 45 min. The primary antibodies were added onto the membranes and incubated at 4 °C overnight. Then, the membranes were washed with TBST for three times (7 min). The membranes were further incubated with specific horseradish peroxidase (HRP)-linked secondary antibodies for 2 h at 25 °C. Finally, the signals from specific proteins were detected using chemiluminescent substrates. The analyst/PC densitometric software (Bio-Rad Laboratory, Hercules, CA, USA, version 6.0.1, 2017) was used to quantify the density of protein signal.

### Statistical analysis

All the data were averaged from three independent experiments. Statistical data analysis was carried out by one-way ANOVA and Tukey HSD post hoc test using SPSS Statistic 22 version (Armonk, NY, USA). Statistical significance was considered at *p* < 0.05.

## Supplementary Information

Supplementary Information

## Data Availability

All data generated or analyzed during this study are included in this article.
