# Supplementary material for: Chemosensitizing activity of peptide from Lentinus squarrosulus (Mont.) on cisplatin-induced apoptosis in human lung cancer cells
Source: Sci Rep. 2021 Feb 18;11:4060. doi: 10.1038/s41598-021-83606-1 (PMC7892851; doi:10.1038/s41598-021-83606-1)
Supplement: Supplementary file 1 — Supplementary Information [file 41598_2021_83606_MOESM1_ESM.pdf]

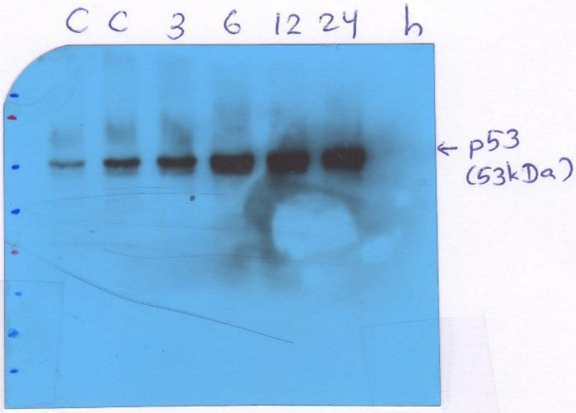


p53

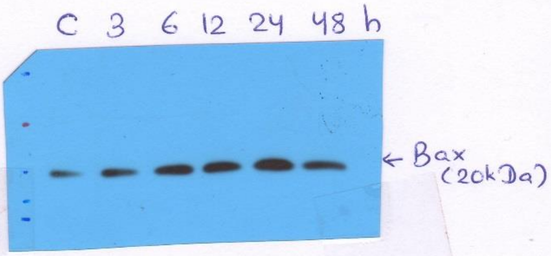


Bax

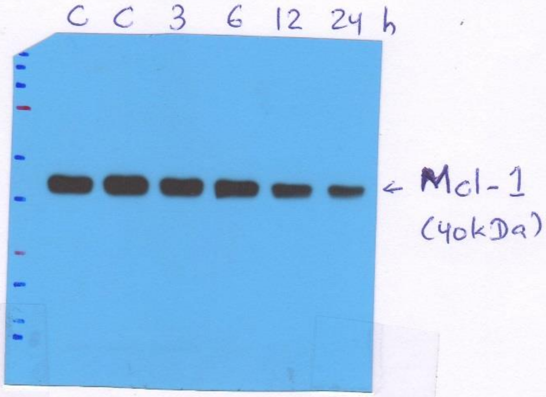


Mcl-1

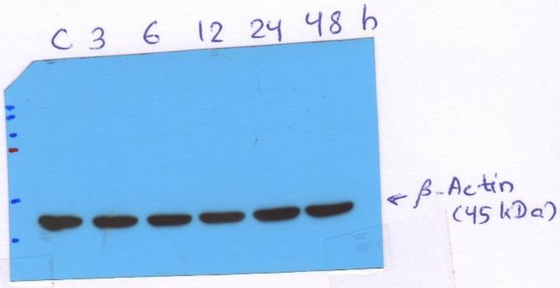


β-Actin

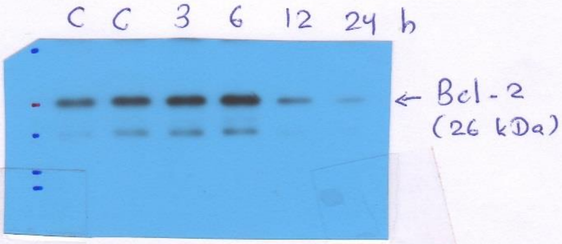


Bcl-2

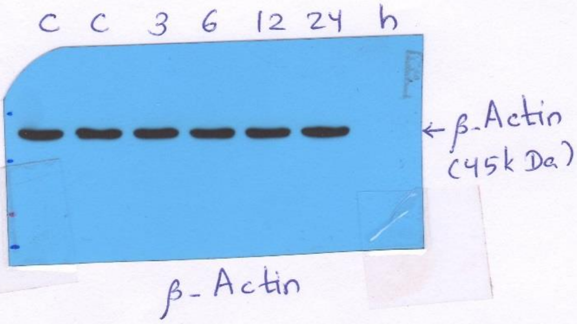


β-Actin

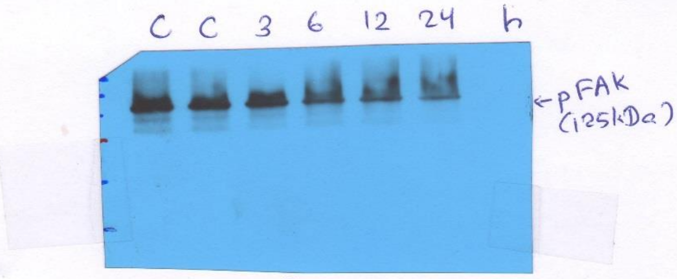


pFAK

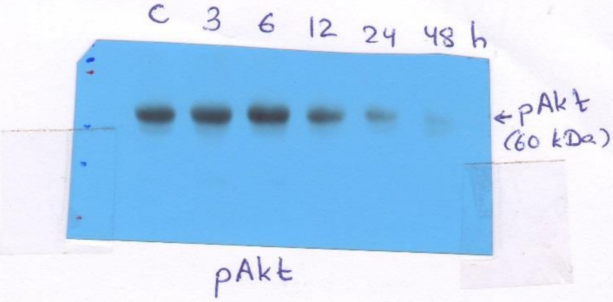


pAkt

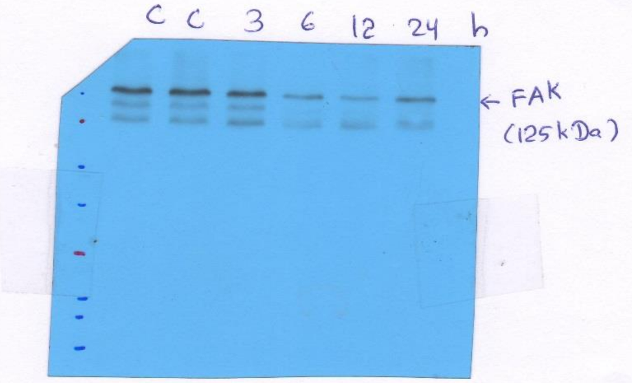


FAK

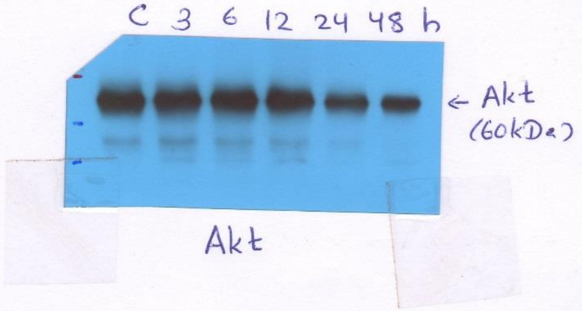


Akt

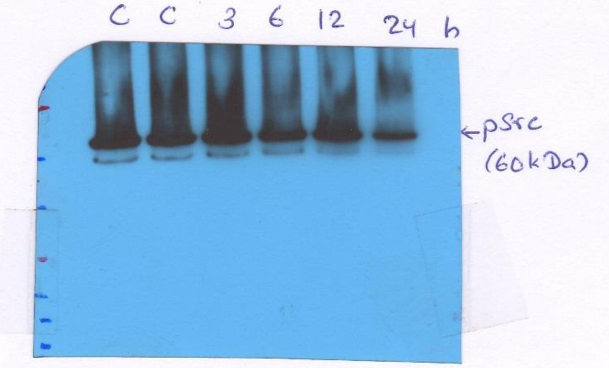


pSrc

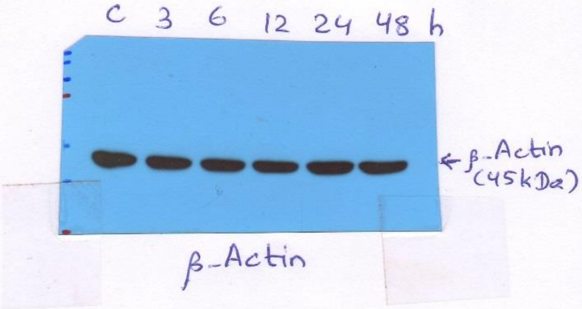


β-Actin

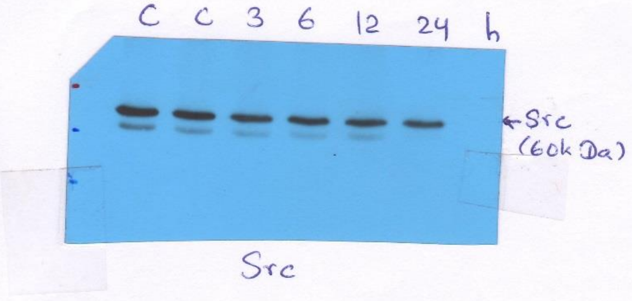


Src

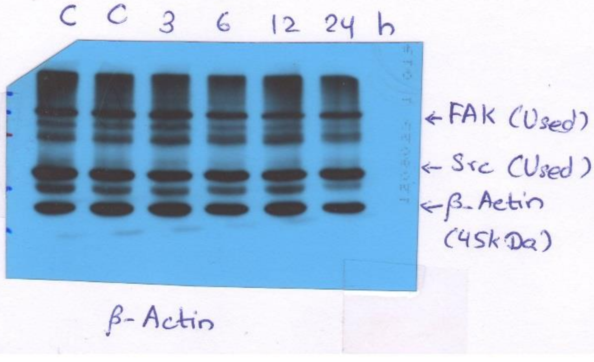


β-Actin

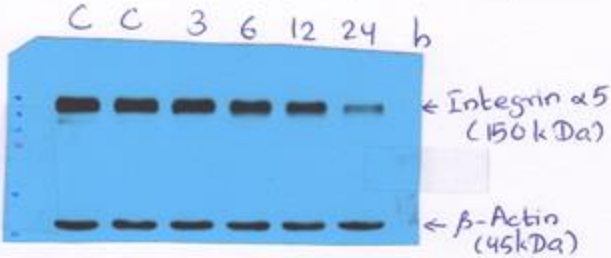


Integrin α5

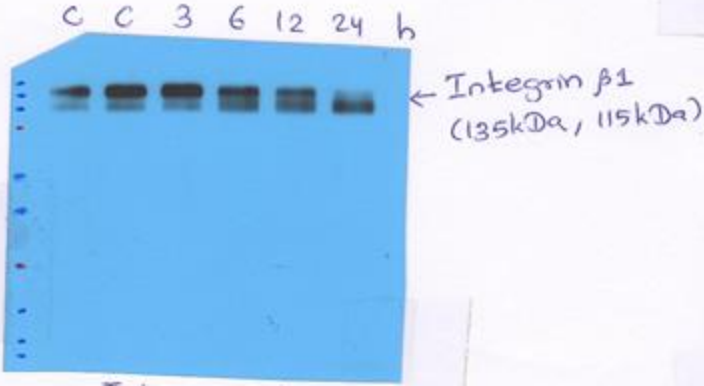


Integrin β1

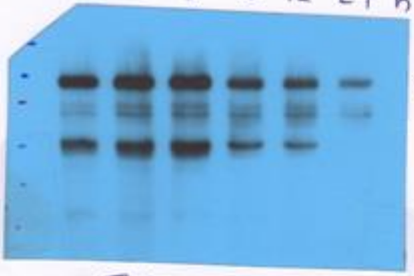


Integrin β3

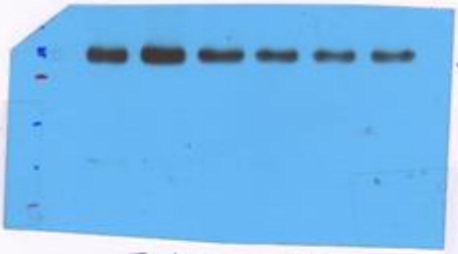


Integrin β5

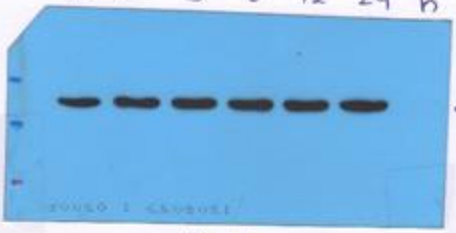


β-Actin

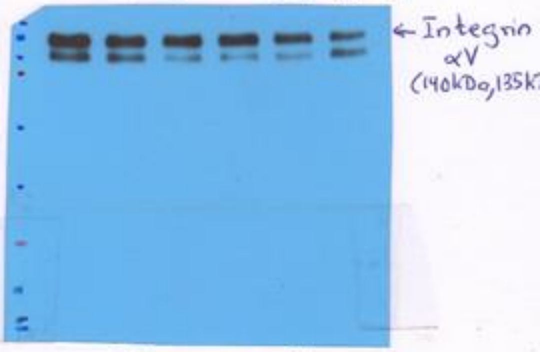


Integrin αV

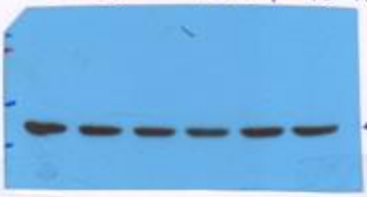


β-Actin

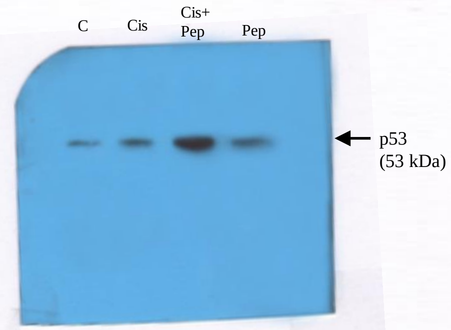


**p53**

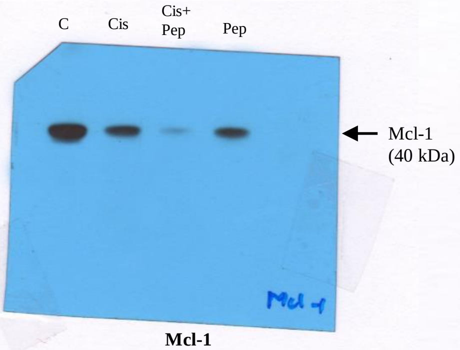


**Mcl-1**

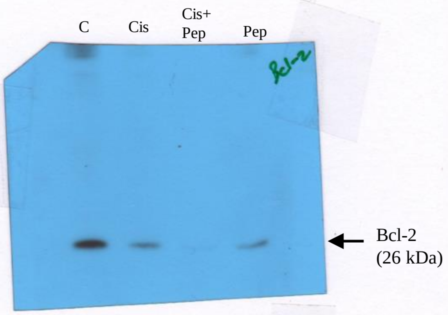


**Bcl-2**

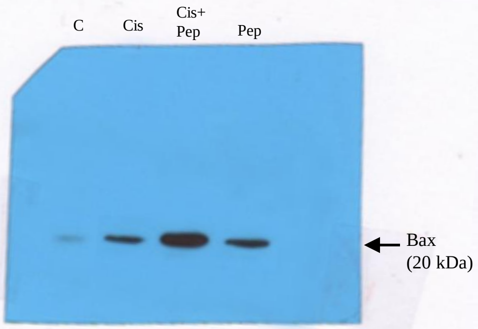


**Bax**

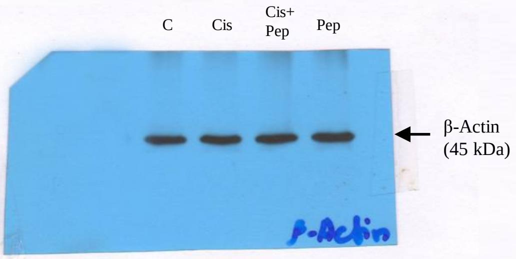


**β-Actin**

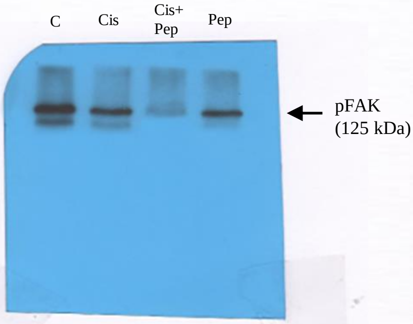


**pFAK**

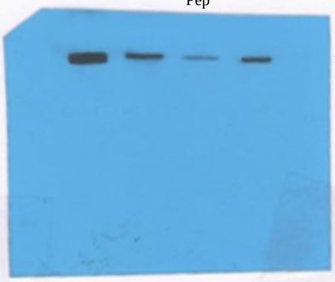


**FAK**

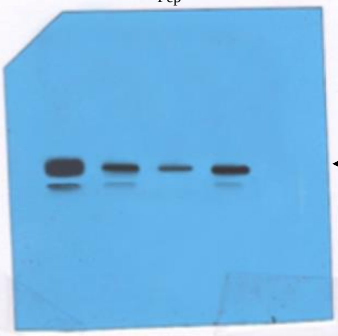


**pSrc**

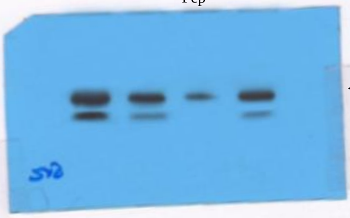


**Src**

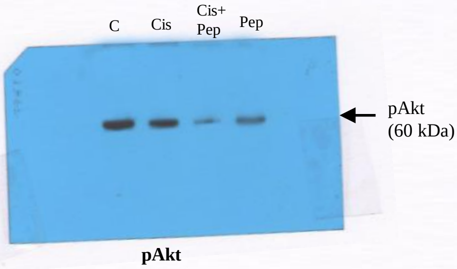


**pAkt**

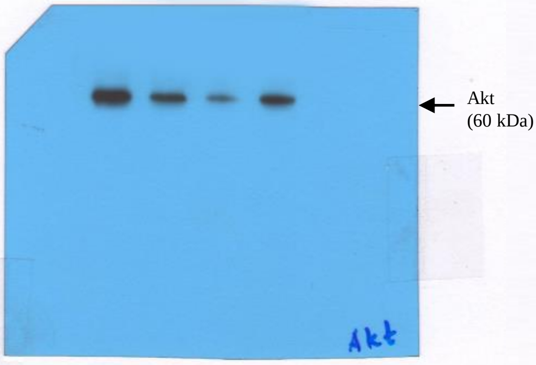


**Akt**

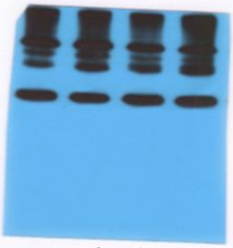


**β-Actin**

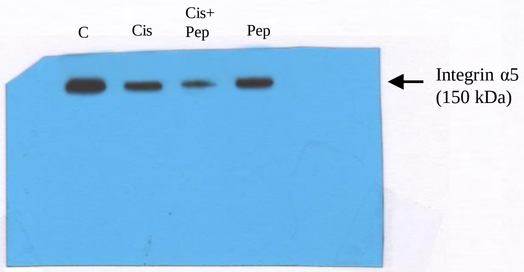


**Integrin α5**

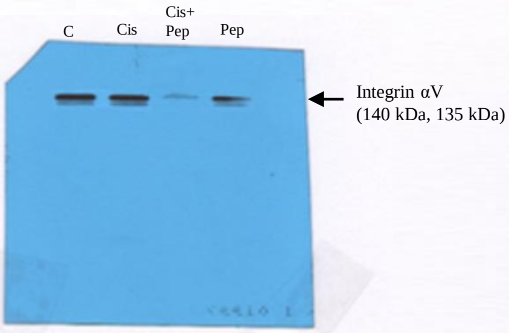


**Integrin αV**

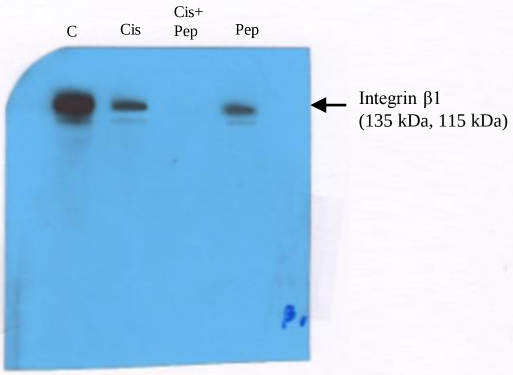


**Integrin β1**

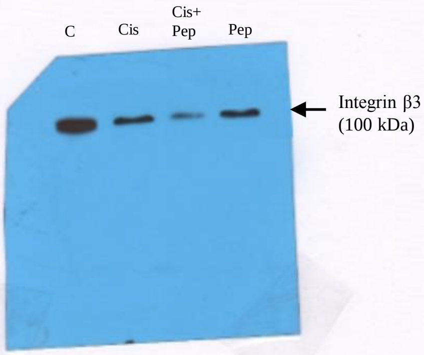


**Integrin β3**

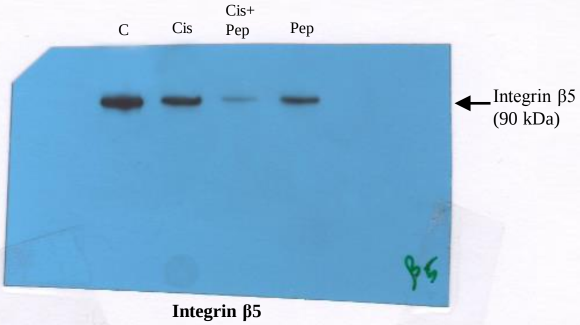


**Integrin β5**

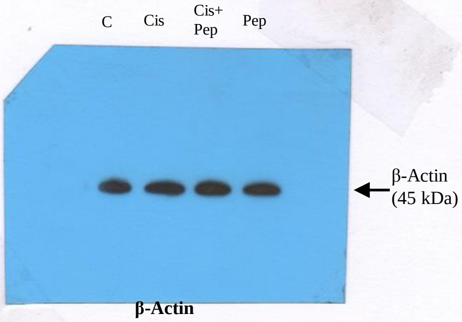


**β-Actin**
